# Well Worthy of Attention

**Published:** 1903-01

**Authors:** 


### Well Worthy of Attention.

At this season of the year the interest of the medical profession in the treatment of inflammation of the respiratory organs is evidenced by the large amount of space devoted to this subject by the medical journals. It would seem that such an exceedingly common-place subject as the ordinary cold, worn almost thread-bare by centuries of discussion, would offer little attraction to the modern physician; yet it is a theme that receives constantly the attention of many of the leaders in medicine. The reason for this can be found in two well recognized facts. First, that an improperly treated acute inflammation of the respiratory organs is often the percursor of grave pathologic conditions that influence other parts of the body and are difficult if not impossible to permanently overcome; an instance of this is the condition of emphysema that often results from a chronic cough, which in turn leads to dilatation of the heart with its concomitant changes in the heart muscles. Second, the treatment of acute inflammations of the respiratory tract by the ordinary employed methods is usually unsatisfactory. That expectorants and cough sedatives are of but little utility is the consensus of opinion of the best men; that they may in a certain limited class of cases be of some value is recognized, but it is also conceded that in the great majority of cases their effects are comparable to that of water on a duck's back. The same may be said of the much-employed cough sedatives of which opium morphine, or one of its derivatives are the most conspicuous examples. It is true that this latter group of agents have a tendency to reduce the frequency and severity of cough, but the principal effects of opium and its derivatives consist of deleterious influences on other physiologic functions; so true is this that the most discriminating of clinicians reserve opium for scattered isolated cases. If a physician would call to the mind the pathologic conditions present in respiratory inflammation it would give a clue as to the best method of treatment that would be consistent with the laws of nature. For instance, in *acute bronchitis*—by far the most common respiratory affection—the mucous membrane is congested, swollen, and because of disturbances of physiologic function, covered with the products of disordered secretion, i. e., mucus or the products of its decomposition or chemical change. These disordered products act just as a foreign body in any other part of the body acts—it produces irritation which manifests itself as cough, and the usual well-known sensations. What more appropriate agent could be applied to this condition of congestion, irritation, hyperæsthesia, and abrogation of function than a remedy that is sedative, demulcent and lubricant? A priori, Petroleum would seem to be the ideal agent, and Angier's Petroleum Emulsion the form to administer it in a pure, palatable and most efficacious form. Aside from the above mentioned theoretical reasons for the use of petroleum in acute respiratory inflammation, there exists the incontrovertible clinical fact that it has for many years yielded better and quicker results in these cases than any other remedy or combination of drugs known. It is, first of all, harmless and entirely free from detrimental influences upon any function or organ of the body. Expectorants, cough syrups and sedatives have a predeliction for irritating the gastric mucous membrane and thereby causing nausea, oft-times vomiting and almost always disturbances of digestion. In contrast to this petroleum has a positive sedative influence on the gastro-intestinal tract. If there were no other recommendations for the use of petroleum in respiratory inflammations, the immediate effects of the remedy in affording relief from the bronchial distress, hacking cough and difficult expectoration would entitle it to the universal use as a palliative. But it is more than this; there are very few cases of acute inflammation of the bronchi, larynx and pharynx that will not be completely eradicated within a week or so after the administration of the remedy has been started. Patients always comment upon the comfortable feeling of the throat and chest after they begin to take petroleum; this means simply that the rawness and soreness due to the congested irritable condition of the mucous membrane have been overcome by the well-attested sedative, demulcent and lubricant properties of the petroleum.

Few, if any, acute cases will become chronic if petroleum is employed, as soon as the first symptoms appear; but chronic cases form a large part of the physician's winter work and are usually intractable and distressing. In chronic bronchitis of the various clinical forms and particularly in that class designated "winter cough" petroleum has achieved an enviable reputation. Because of its above mentioned local effects it is of specific value in overcoming the morbid secretory disturbances of the bronchial mucous membrane which constitutes the essential feature of these chronic cases. That it has this effect is proved by the diminution in the frequency and severity of the cough, the alteration of the expectorated material from a thick viscid, tenacious mass to a fluid easily expelled, less copious, mucoid material and the freedom afforded the patient from the subjective symptoms of irritation in the thorax. In elderly people with chronic winter cough of yearly recurrence and of obstinate character, the use of strychnine in combination with petroleum yields results far better than any other method of treatment. Probably not a small element of the success of petroleum in chronic respiratory inflammation is due to the positive effects of the remedy as a nutritive; it will be recalled that many of the obstinate cases are associated with or even dependent upon constitutional conditions of malnutrition or general debility. As a nutritive, petroleum is, authorities of the highest class have proved, far superior to Cod Liver Oil. This fact has been demonstrated beyond a question of doubt, and is accepted as one of the established facts of therapeutics.

In view of what many years of experiences of carefully observant physicians have proved, it may be safely stated that the treatment of both acute and chronic bronchitis and other inflammations of the respiratory tract, will be uniformly satisfactory if petroleum is administered as soon as the condition begins and continued until convalescence is firmly established.

